# Perfusion MR imaging detection of carcinoma arising from preexisting salivary gland pleomorphic adenoma by computer-assisted analysis of time-signal intensity maps

**DOI:** 10.1371/journal.pone.0178002

**Published:** 2017-05-22

**Authors:** Ikuo Katayama, Sato Eida, Shuichi Fujita, Yuka Hotokezaka, Misa Sumi, Takashi Nakamura

**Affiliations:** 1 Department of Radiology and Cancer Biology, Nagasaki University Graduate School of Biomedical Sciences, Nagasaki, Japan; 2 Department of Oral Pathology and Bone Metabolism, Nagasaki University Graduate School of Biomedical Sciences, Nagasaki, Japan; Charles P. Darby Children's Research Institute, 173 Ashley Avenue, Charleston, SC 29425, USA, UNITED STATES

## Abstract

Tumor perfusion can be evaluated by analyzing the time-signal intensity curve (TIC) after dynamic contrast-enhanced (DCE) MR imaging. Accordingly, TIC profiles are characteristic of some benign and malignant salivary gland tumors. A carcinoma ex pleomorphic adenoma (CXPA) arises from a long-standing pleomorphic adenoma (PA) and has a distinctive prognostic risk depending on the tumor growth potential such as invasion beyond the preexisting capsule. Differentiating CXPA from PA can be very challenging. In this study, we have attempted to discriminate CXPA from PA based on a two-dimensional TIC mapping algorithm. TIC mapping analysis was performed on 8 patients with CXPA and 20 patients with PA after dynamic contrast-enhanced (DCE) MR imaging using a 1.5-T MR system. The TIC profiles obtained were automatically categorized into 5 types based on the enhancement ratio, maximum time, and washout ratio (Type 1 TIC with flat profile, Type 2 TIC with slow uptake, Type 3 TIC with rapid uptake and a low washout ratio, Type 4 TIC with rapid uptake and a high washout ratio, and Type 5 TIC not otherwise specific). The percentage tumor areas with each of the 5 TIC types were compared between CXPAs and PAs. Stepwise differentiation and cluster analysis using multiple TIC cut-off thresholds distinguished CXPAs from PAs with 75% sensitivity, 95% specificity, 86% accuracy, and 86% positive and 90% negative predictive values, when tumors with ≤1.1% Type 1 and ≥15% Type 4, or those with ≤1.1% Type 1, ≥78.1% Type 2, ≥16.1% Type 3, and <15% Type 4, or those with >1.1% Type 1, ≥78.1% Type 2, and ≥16.1% Type 3 areas were diagnosed as CXPAs. The overall TIC profiles predicted some aggressive CXPA growth patterns. These results suggest that stepwise differentiation based on TIC mapping is helpful in differentiating CXPAs from PAs.

## Introduction

Tumor perfusion can be readily evaluated by analyzing the time-signal intensity curve (TIC) after dynamic contrast-enhanced (DCE) MR imaging. Accordingly, TIC profiles are characteristic of some benign and malignant tumors. In fact, previous studies have confirmed the usefulness of TIC profile assessment in differentiating between benign and malignant salivary gland tumors [1].

Carcinoma ex pleomorphic adenoma (CXPA) arises from its long-standing benign counterpart, pleomorphic adenoma (PA), probably due to persistent genomic instability conditions leading to gene arrangement/amplification, with progressive involvement of chromosomal arms 8q, 12q, and then 17p [2, 3]. Rapidly growth with or without pain and facial nerve palsy may be associated with CXPA [4]. However, preoperative discrimination of CXPA from PA can be very challenging. The proportion of malignant components in the tumor can be quite variable, and it may occupy a small part of the background PA, which consists of heterogeneous histological components including proliferating tumor cells, myxomatous tissues, non-enhanced areas such as necrotic tissues/cysts [4]. CXPAs may be very invasive with varying extents of cancer cell invasion beyond the tumor capsule. The invasive type of CXPA has a poorer prognosis than the non-invasive type [4–7]. Therefore, preoperative differentiation of CXPA from PA is very helpful in planning patient management.

Additional information on tumor perfusion other than conventional MR imaging findings can facilitate the effective differentiation of CXPA from PA. However, TIC analysis using a large region of interest (ROI) may result in spurious results owing to the considerably overlapping histological features of CXPA and PA. Recent studies have successfully applied pixel-based TIC analysis for differentiating between benign and malignant head and neck tumors, including salivary gland tumors [8, 9]. In the pixel-based TIC analysis, the ultimate ROI is a single pixel. Therefore, by analyzing the elemental TICs, which represent the changes in contrast agent concentration with time in each pixel, changes in perfusion associated with malignant transformation within varying extents of the pre-existing benign counterpart can be identified. Therefore, we have attempted to differentiate CXPA from PA based on a two-dimensional TIC mapping algorithm.

## Materials and methods

### Patients

We retrospectively analyzed dynamic contrast-enhanced (DCE) MR images obtained from 8 patients with carcinoma ex pleomorphic adenoma (CXPA; 3 women and 5 men; average age, 64 years, age range 54–82 years) and 20 patients with pleomorphic adenoma (PA; 16 women and 4 men; average age, 57 years, age range, 19–90 years), who had undergone preoperative MR examinations at our hospital between 2003 and 2012. The other inclusion criteria were as follows: (a) Tumors had large sizes (>10 mm in short-axis diameters) adequate for analyses (including necrotic/cystic components), (b) Tumors were excised and histologically diagnosed, and (c) Tumor MR images were good in quality without severe susceptibility or motion artifacts. We excluded 1 patient from the study because of insufficient image quality due to motion artifacts. This retrospective study was approved by the Nagasaki University Hospital Ethics Committee. Informed consent was waived off because of the retrospective nature of this study.

The locations for CXPAs were the palate (n = 3), parotid gland (n = 1), submandibular gland (n = 1), parapharyngeal space (n = 1), tongue (n = 1), and oral floor (n = 1). For PAs, the locations were the submandibular gland (n = 9), parotid gland (n = 6), palate (n = 3), parapharyngeal space (n = 1), and buccal space (n = 1) for PAs.

Histological subtypes of malignant components within the CXPAs included adenocarcinomas (n = 4), adenoid cystic carcinoma (n = 1), undifferentiated carcinomas (n = 1), myoepithelial carcinoma (n = 1), mucoepidermoid carcinoma (n = 1), polymorphous low-grade adenocarcinoma (n = 1), and salivary duct carcinoma (n = 1). A single case with CXPA had 3 different malignant histological subtypes (adenocarcinoma, adenoid cystic carcinoma, and polymorphous low-grade adenocarcinoma).

### MR imaging

MR imaging was performed using a 1.5-T MR imager (Gyroscan Intera 1.5T Master, Philips Healthcare, Best, The Netherlands) with a 140 mm × 170 mm Synergy Flex M coil (Philips Healthcare). T1- and fat-suppressed (spectral attenuated with inversion recovery, SPAIR) T2-weighted MR images (TR/TE/number of signal acquisition = 500 ms/15 ms/2, and 6385 ms/80 ms/2, respectively) were obtained by using a turbo spin-echo (TSE) sequence (TSE factor = 3 and 15, respectively). We used a 200-mm FOV, 256 × 204 acquisition and 512 × 512 reconstruction matrix sizes, a 4-mm slice thickness and a 0.4-mm slice gap. For DCE studies, axial T1-weighted images (TR/TE/number of signal acquisitions = 306 ms/10 ms/1) were obtained using a TSE sequence (TSE factor = 7), 200-mm field of view (FOV), 4- to 5-mm slice thickness, 0.4- to 0.5-mm slice gap, and a 256 × 180 matrix size. Gadolinium (gadopentetate dimeglumine, Magnevist, Bayer HealthCare) was injected intravenously at a dose of 0.2 ml/kg body weight and at an injection rate of 1.5 ml/s, followed by a 20-ml saline flush. In each patient, 19 MR data-acquisitions were obtained at an interval of 10 s (0 ~ 180 s). We analyzed 3 slices from the mid-portion of each tumor. The MR images used in the study were assessed from the Nagasaki University Hospital database and the patient data were anonymised.

### Conventional MR image characterization

T1- and fat-suppressed T2-weighted MR images of CXPAs and PAs were evaluated by 2 radiologists with 17 and 22 years of experience in the field of head and neck radiology. Consensus reading of the images was based on the signal properties of tumor parenchyma (homogeneous or heterogeneous relative to the neighboring muscle on fat-suppressed T2-weighted MR images) and on the signal characteristics of the tumor capsule (the presence or absence of contrast-enhanced tumor demarcation surrounding the entire tumor perimeter at the maximum tumor area on CE T1-weighted MR images).

### Time signal intensity curve (TIC) analysis

The sequential dynamic MR images in a Digital Imaging and Communications in Medicine (DICOM) format were transferred onto a personal computer and were then analyzed by using the ImageJ (NIH, https://imagej.nih.gov/ij/) and Mathematica (Wolfram Research) softwares. In this analysis system, an ROI was manually placed onto a tumor area to include as much tumor area as possible (not excluding necrotic/cystic parts) on a pre-enhanced (= 0 s) T1-weighted MR image, and it was automatically placed onto the subsequent 18 dynamic MR images by repeated copying and pasting the initial ROI using the ImageJ software. Subsequently, the tumor areas were extracted using the ImageJ software. The TIC analysis was performed by using the Mathematica software on a pixel-by-pixel basis (TIC mapping) as follows.

**Fig 1:**
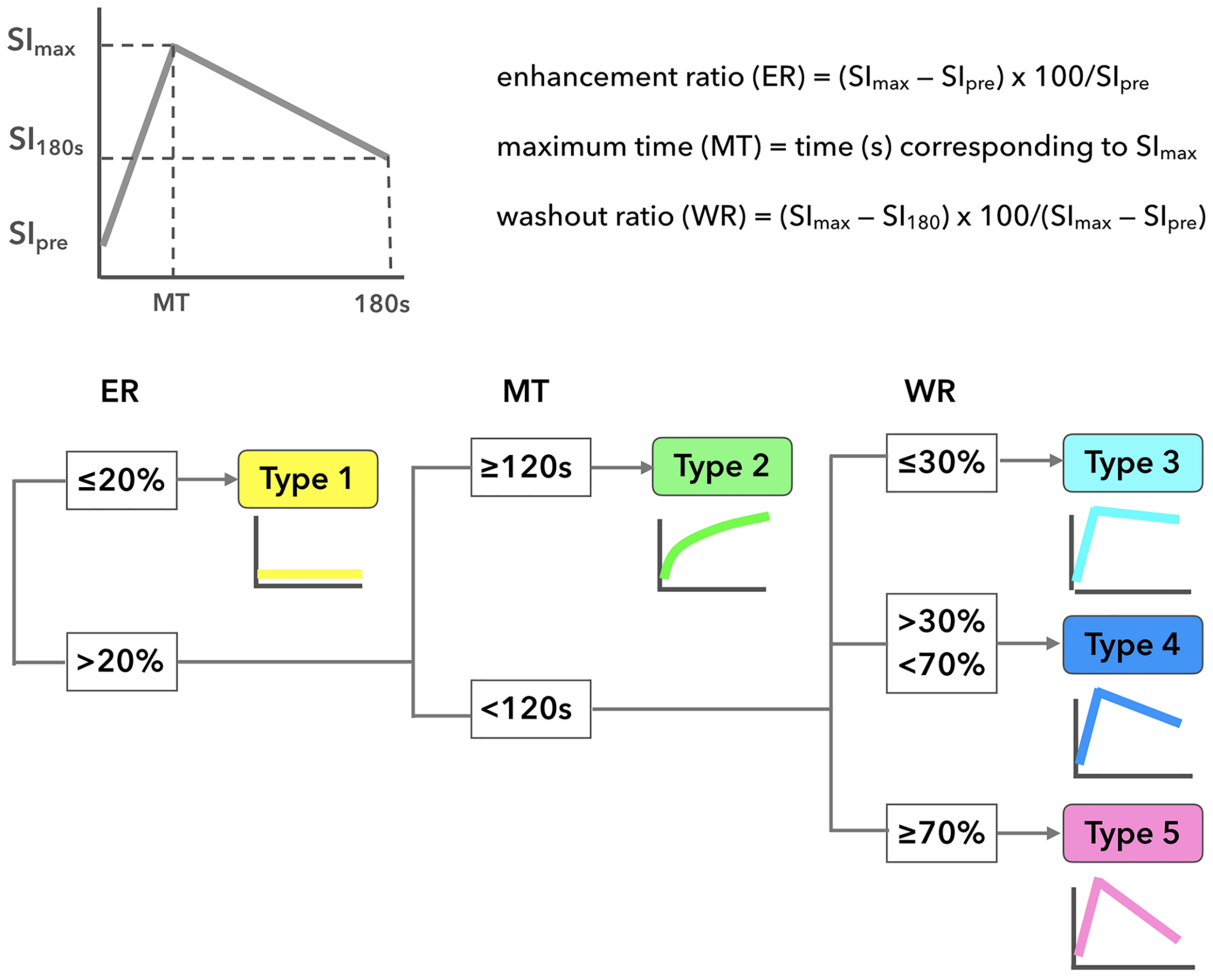
Classification of time-signal intensity curves (TICs) after dynamic contrast-enhanced (DCE) MR imaging. Upper panel shows definitions of TIC parameters: enhancement ratio (ER), maximum time (MT), and washout ratio (WR). The TICs obtained were classified into five types (Types 1–5) based on the ER, MT, and WR values (lower panel).

Each of the obtained TICs were automatically classified into 5 types (Types 1–5) based on the enhancement ratio (ER), maximum time (MT), washout ratio (WR) (Fig 1). These TIC parameters were calculated using the following equations: ER (%) = (maximum signal intensity – pre-enhancement signal intensity) × 100/signal intensity of pre-enhancement; MT = time corresponding to the maximum signal intensity; WR (%) = (maximum signal intensity – signal intensity 180s after the start of contrast medium injection) × 100 /(maximum signal intensity – pre-enhancement signal intensity).

Type 1 TICs are those with ERs smaller than or equal to 20%. Type 2 TICs are those with ERs greater than 20% and MTs equal to or longer than 120 s, Type 3 TICs are those with ERs greater than 20%, MTs shorter than 120 s, and WRs smaller than or equal to 30%. Type 4 TICs are those with ERs greater than 20%, MTs shorter than 120 s, and WRs greater than 30% and smaller than 70%. Type 5 TICs are those with ERs greater than 20%, MTs shorter than 120 s, and WRs equal to or greater than 70% (Fig 1). Accordingly, the TIC patterns were referred to flat (Type 1), slow uptake (Type 2), rapid uptake with a low WR (Type 3), rapid uptake with a high WR (Type 4), or not otherwise specified (Type 5).

The percentage areas corresponding to each of the 5 TIC patterns of the total tumor area (a sum of 3 sequential tumor areas including the maximum tumor area) were calculated, and the two-dimensional distributions of the distinct TIC patterns in the 3 sequential tumor areas were displayed as color TIC maps.

Previous studies have shown that TIC profiles correlate well with the histological components of the tissues [10]. Accordingly, we tentatively categorized the overall TIC profiles of the CXPAs and PAs into 4 types, referring to them as matrix-rich, cell-rich, intermediate, and cystic types. The matrix-rich type indicated tumors with ≥60% Type 2 areas, the cell-rich type had ≥15% Type 4 TIC areas, the intermediate type had <60% Type 2 and <15% Type 4 areas, and the cystic type contained ≥50% Type 1 areas.

### Histological assessment of CXPAs

Histologic characteristics of 8 CXPAs were analyzed using hematoxylin- and eosin-stained paraffin-embedded tissues from the mid-portion of the excised tumors. These were based on the presence (invasive) or absence (non-invasive) of extensive tumor invasion (≥1.5 cm cancer extension beyond the tumor capsule), histological subtypes of the malignant components (adenocarcinoma, adenoid cystic carcinoma, undifferentiated carcinoma, salivary duct carcinoma, etc.), and the proportion of malignant components within the tumors expressed as a percentage of the total tumor area [11]. Subsequently, we correlated the histological characteristics with the overall TIC profiles of the tumors. TIC profiles were also compared between the pre-existing PA and malignant tumor areas by analyzing the TIC map areas that roughly corresponded to either of the tumor areas within the 8 CXPAs.

### Statistical analysis

Statistical analysis was performed using the JMP software (version 11, SAS, Chicago, IL). TIC cut-off thresholds for differentiating between CXPAs and PAs were determined using receiver operating characteristic (ROC) analysis, followed by cluster analysis to perform a stepwise differentiation of CXPA from PA using multiple TIC criteria. Average percent tumor areas with distinctive TIC patterns of CXPA and PA were compared using the Welch’s t-test. The sensitivity, specificity, accuracy, and positive and negative predictive values for the single or multiple cut-off thresholds to diagnose CXPA or PA were studied. For all comparisons, a value of p<0.05 was considered statistically significant.

## Results

### Conventional MR imaging findings of CXPAs and PAs

**Fig 2:**
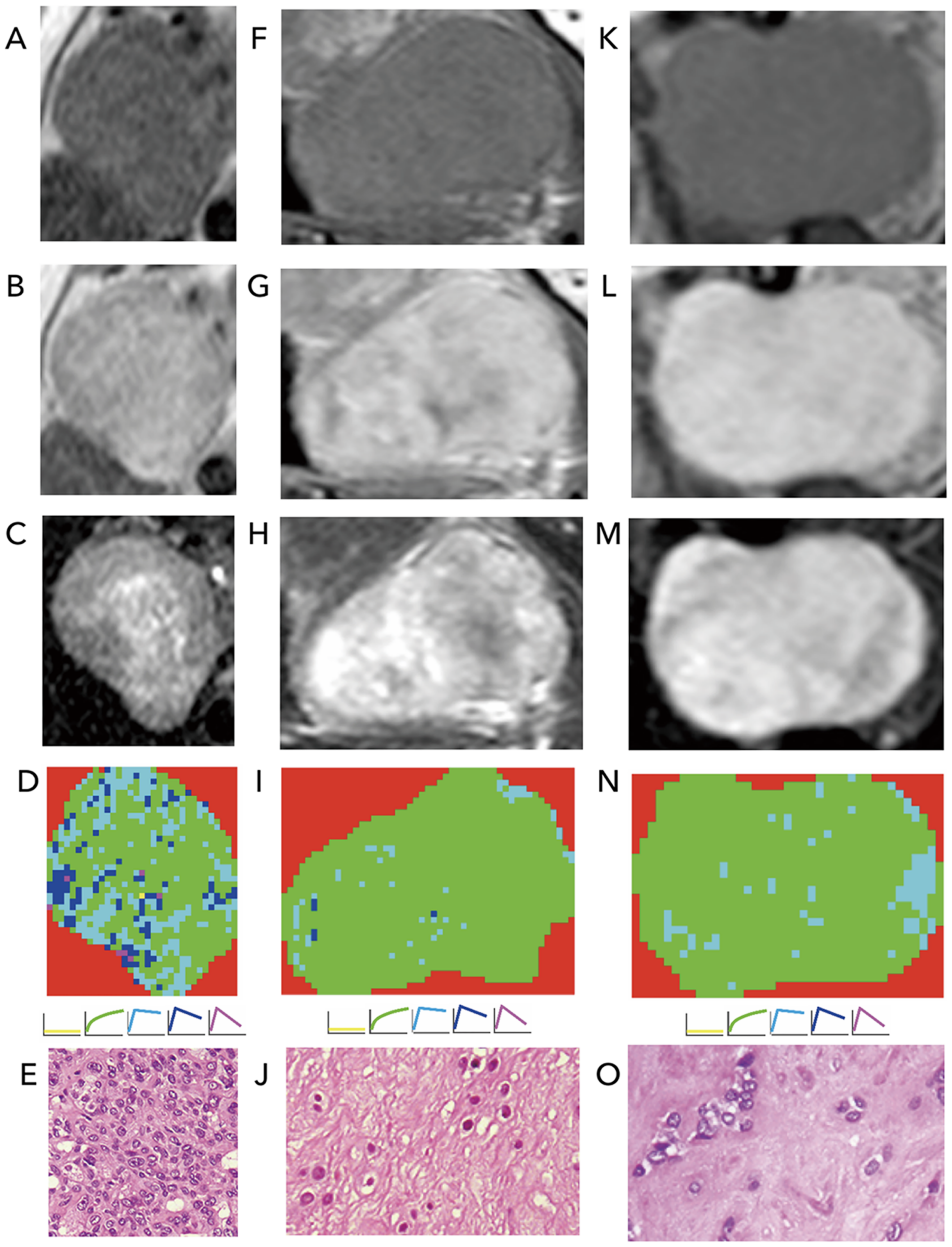
Conventional MR imaging and TIC analysis. **A**-**E**, 72-year-old man with carcinoma ex pleomorphic adenoma (CXPA) in right submandibular gland. Tumor is homogeneous (**A**), irregularly enhanced (**B**), and heterogeneous (**C**) on T1 (T1WI)-, contrast-enhanced T1 (CET1WI)-, and fat-suppressed T2-weighted (fsT2WI) images, respectively. TIC mapping indicates tumor consisting of 0.2% Type 1, 57.2% Type 2, 26.9% Type 3, 15% Type 4, and 0.8% Type 5 (overall TIC profile = cell-rich type, **D**). Photomicrograph shows tumor area with high cellularity (**E**, H & E staining). Tumor was correctly diagnosed by stepwise differentiation algorithm. **F**-**J**, 61-year-old man with CXPA in left parapharyngeal space. Tumor is homogeneous (**F**), irregularly enhanced (**G**), and heterogeneous (**H**) on T1WI, CET1WI, and fsT2WI, respectively. TIC mapping indicates tumor consisting of 2.8% Type 1, 93.4% Type 2, 2.6% Type 3, 0.8% Type 4, and 0.4% Type 5 (overall TIC profile = matrix-rich type, **I**). Photomicrograph shows tumor area rich in fibro-hyalinous tissues (**J**, H & E staining). Tumor was misdiagnosed by stepwise differentiation algorithm. **K**-**O**, 34-year-old man with PA in left parotid gland. Tumor is homogeneous (**K**), irregularly enhanced (**L**), and heterogeneous (**M**) on T1WI, CET1WI, and fsT2WI, respectively. TIC mapping indicates tumor consisting of 0.0% Type 1, 94.2% Type 2, 5.8% Type 3, 0.0% Type 4, and 0.0% Type 5 (overall TIC profile = matrix-rich type, **N**). Photomicrograph shows tumor area rich in fibromyxoid tissues (**O**, H & E staining). Tumor was correctly diagnosed by stepwise differentiation algorithm.

Findings in terms of the presence or absence of a contrast-enhanced capsule that surrounds the entire tumor perimeter, and of the homogeneous or heterogeneous tumor signal characteristics are summarized in Table 1. We found that both the MR imaging criteria were not helpful for differentiating between the two tumors (Fig 2A–2C, 2F–2H and 2K–2M).

**Table 1:** Conventional MR imaging findings of 8 CXPAs and 20 PAs.

	n	complete capsule on CET1WI* (%)		tumor parenchyma on fsT2WI** (%)	
		+	—	homogeneous	heterogeneous
CXPA	8	5 (63)	3 (38)	0 (0)	8 (100)
PA	20	13 (65)	7 (35)	0 (0)	20 (100)
p-value***		1.432		NS	

CXPA, carcinoma ex pleomorphic adenoma; PA, pleomorphic adenoma; CET1WI, contrast-enhanced T1-weighted MR image; fsT2WI, fat-suppressed T2-weighted MR image

*, A complete capsule was defined as an enhanced demarcation of the entire perimeter of a tumor on CET1WI that included the maximum tumor area.

**, Tumor parenchyma was categorized into homogeneous or heterogeneous relative to the neighboring muscle(s) on fsT2WI.

***, chi-square test (two-tailed). NS, not significant

### Diagnostic performance of single TIC criteria

Detailed TIC profiles of the 8 patients with CXPA and 20 patients with PA are shown in Table 2. The average percent tumor areas with distinctive TIC patterns (Type 1-Type 5) were not significantly different between CXPAs and PAs. ROC analysis indicated that ≤1.1% Type 1 tumor area, ≥78.1% Type 2 area, ≥16.1% Type 3 area, ≥15.0% Type 4 area, and ≥10% Type 5 tumor area were the best cut-off thresholds for differentiating CXPAs from PAs (Table 3 and Fig 2D, 2I and 2N). However, the single TIC cut-off thresholds provided low or at best moderate diagnostic abilities for differentiating between the two salivary gland tumor entities.

**Table 2:** Tumor locations, TIC profiles, and results of stepwise analysis of 8 CXPAs and 20 PAs.

patient	locus	TIC profile (%)						Stepwise
								analysis
		Type 1	Type 2	Type 3	Type 4	Type 5	overall TIC	
CXPA								
1	OF	0.0	81.6	16.7	1.7	0.0	matrix-rich	TP
2	PL	1.1	44.3	45.1	8.6	0.1	intermediate	FN
3	PL	0.0	78.1	18.8	3.1	0.0	matrix-rich	TP
4	PL	0.1	78.7	17.4	3.9	0.1	matrix-rich	TP
5	PG	0.0	56.9	41.2	1.9	0.0	intermediate	FN
6	SMG	0.2	57.2	26.9	15.0	0.8	cell-rich	TP
7	PPS	2.8	93.4	2.6	0.8	0.4	matrix-rich	FN
8	TG	0.0	51.0	16.1	23.0	9.9	cell-rich	TP
average*		0.5 ± 1.0	67.7 ± 17.5	23.1 ± 14.1	7.3 ± 7.9	1.4 ± 3.4		
PA								
9	PL	2.5	73.8	15.2	7.4	1.2	matrix-rich	TP
10	PL	0.0	71.9	26.0	2.0	0.0	matrix-rich	TP
11	PL	2.3	80.6	13.0	3.9	0.2	matrix-rich	TP
12	PG	0.0	94.2	5.8	0.0	0.0	matrix-rich	TP
13	PG	60.1	26.2	8.1	4.7	0.8	cystic	TP
14	PG	0.0	47.9	41.0	10.8	0.3	intermediate	TP
15	PG	0.0	85.6	13.0	1.4	0.0	matrix-rich	TP
16	PG	0.0	63.2	23.7	9.2	3.9	matrix-rich	TP
17	PG	0.0	47.2	47.3	5.3	0.2	intermediate	TP
18	SMG	7.9	53.5	16.5	10.4	11.7	intermediate	TP
19	SMG	0.2	69.5	20.1	10.0	0.2	matrix-rich	TP
20	SMG	12.5	62.4	17.7	6.0	1.4	matrix-rich	TP
21	SMG	0.0	72.2	25.2	2.6	0.0	matrix-rich	TP
22	SMG	0.7	86.7	9.7	2.2	0.7	matrix-rich	TP
23	SMG	1.3	78.0	13.4	6.3	1.0	matrix-rich	TP
24	SMG	2.0	74.0	21.9	2.0	0.1	matrix-rich	TP
25	SMG	3.8	51.9	35.0	7.9	1.4	intermediate	TP
26	SMG	0.0	23.8	57.6	18.6	0.0	cell-rich	FN
27	PPS	0.2	97.8	1.7	0.3	0.0	matrix-rich	TP
28	BS	6.8	55.4	23.8	11.5	2.5	intermediate	TP
average*		5.0 ± 13.1	65.8 ± 20.2	21.8 ± 14.2	6.1 ± 4.7	1.3 ± 2.6		
p-value**		0.147	0.811	0.827	0.715	0.924		

TIC, time-signal intensity curve; CXPA, carcinoma ex pleomorphic adenoma; PA, pleomorphic adenoma; OF, oral floor; PL, palate; PG, parotid gland; SMG, submandibular gland; PPS, parapharyngeal space; TG, tongue; BS, buccal space, TP, true positive; FN, false negative.

Overall TIC profiles were categorized into matrix-rich type (≥60% Type 2 areas), cell-rich type (≥15% Type 4 TIC areas), intermediate type (<60% Type 2 and <15% Type 4 areas), and cystic type (≥50% Type 1 areas).

*, percentage areas with particular TIC types within the total tumor areas (mean ± s.d.)

**, Welch’s t-test

**Table 3:** Diagnostic abilities of single or combined TIC criteria for discriminating CXPAs from PAs.

TIC criteria*				diagnostic ability (%)				
				Sens	Spec	Acc	PPV	NPV
single criteria for CXPA								
≤1.1% Type 1				25	45	39	15	60
≥78.1% Type 2				50	75	68	44	79
≥16.1% Type 3				88	40	54	37	89
≥15% Type 4				25	95	75	67	76
≥9.9% Type 5				13	95	71	50	73
Stepwise approach w/ multiple TIC criteria for CXPA								
1st	2nd	3rd	4th					
≤1.1% Type 1	≥15% Type 4	—	—	75	95	86	86	90
≤1.1% Type 1	<15% Type 4	≥78.1% Type 2	≥16.1% Type 3					
>1.1% Type 1	≥16.1% Type 3	≥78.1% Type 2	—					

TIC, time-signal intensity curve; CXPA, carcinoma ex pleomorphic adenoma; PA, pleomorphic adenoma; 1st, 2nd, and 3rd, 1st, 2nd, and 3rd TIC thresholds for the stepwise differentiation. Sens, sensitivity; Spec, specificity; Acc, accuracy; PPV and NPV, positive and negative predictive value.

*, TIC cut-off thresholds were determined by using ROC analysis.

**, Stepwise approach (1st to 4th) for discriminating CXPA from PA using multiple TIC cut-off thresholds was determined by using cluster analysis.

### Diagnostic performance of multiple TIC criteria in stepwise approach

**Fig 3:**
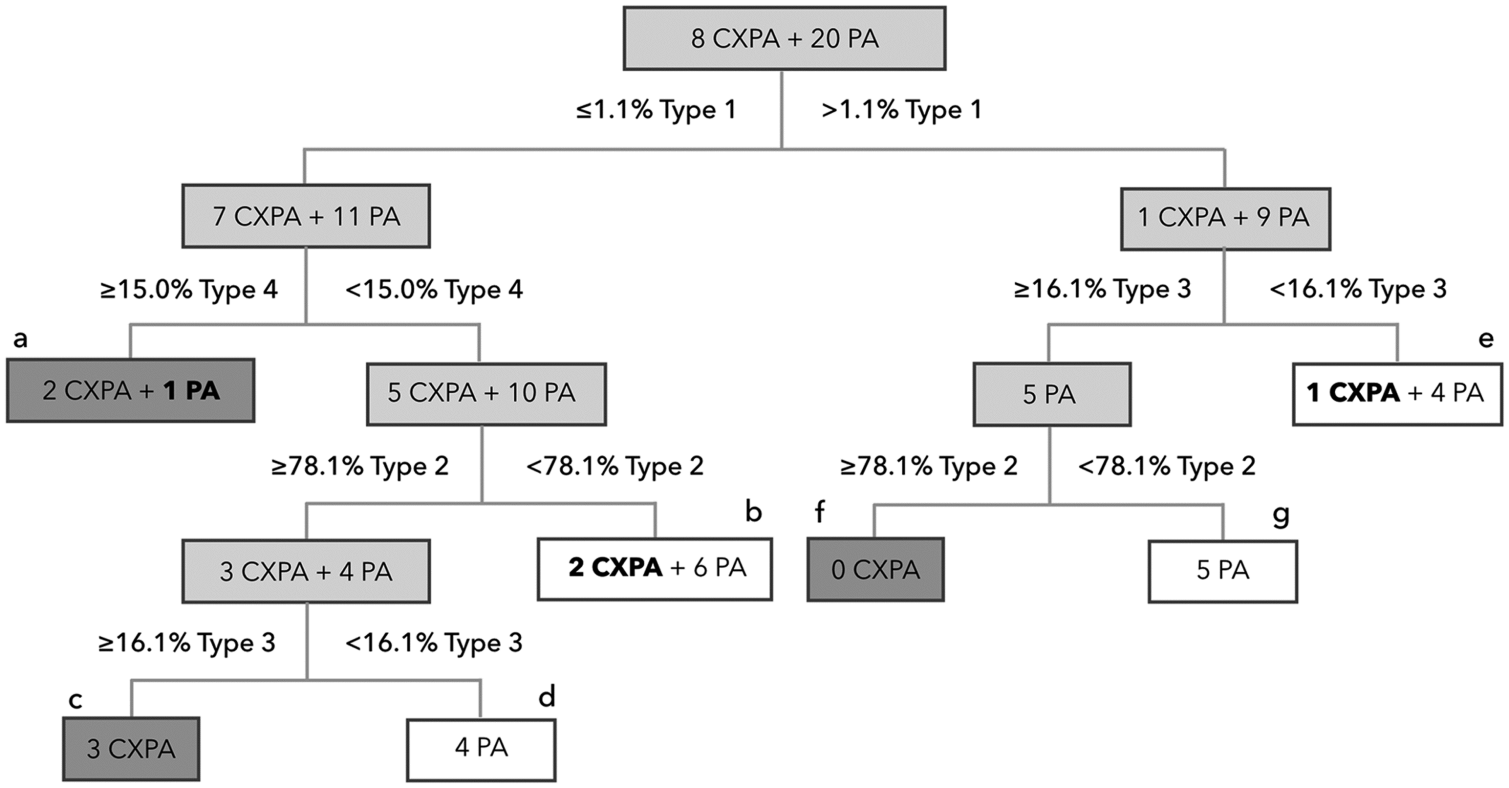
Dendrogram for stepwise differentiation of 8 carcinoma ex pleomorphic adenomas (CXPAs) from 20 pleomorphic adenomas (PAs) using multiple TIC cut-off thresholds. The TIC thresholds were determined by using receiver operating characteristic (ROC) curve analysis. The dendrogram was obtained by using the cluster analysis (JMP version 11). Dark grey (**a**, **c**, and **f**) and open (**b**, **d**, **e**, and **g**) boxes indicate cases that were diagnosed as CXPA and PA, respectively, through the stepwise discrimination. Light grey boxes indicate cases to be diagnosed. Bold numbers and tumor types within the dark grey (one PA in box **a**) and open (two CXPAs in box **b** and one CXPA in box **e**) boxes indicate misdiagnosed (false negative) cases.

We next tested whether combinations of the TIC indices determined by the ROC analysis could improve the diagnostic abilities for differentiating between CXPAs and PAs. To this end, we used a stepwise differentiation strategy with different combinations of TIC cut-off thresholds. Cluster analysis indicated the dendrogram that most effectively differentiated CXPAs from PAs (Fig 3).

The stepwise differentiation strategy could differentiate CXPAs from PAs with 75% sensitivity, 95% specificity, 86% accuracy, and 86% positive and 90% negative predictive values, when tumors with ≤1.1% Type 1 and ≥15% Type 4, or those with ≤1.1% Type 1, ≥78.1% Type 2, ≥16.1% Type 3, and <15% Type 4, or those with >1.1% Type 1, ≥78.1% Type 2, and ≥16.1% Type 3 areas were diagnosed as CXPAs. (Table 3). However, 3 CXPAs and 1 PA were still misdiagnosed using this strategy (Fig 3).

### Correlations of overall TIC profiles and histological features of CXPA

When the tumors were categorized into any of the 3 overall TIC types (matrix-rich, cell-rich, and intermediate types), those with intermediate overall TIC types had the most invasive histological characteristics, such as invasive tumor margins and greater proportions of malignant components within the tumor (Table 4, Fig 2E, 2J and 2O). However, the small study population of CXPA did not allow us to reach a conclusion in terms of statistically significant correlations between the aggressive histological characteristics and the overall TIC profiles.

**Table 4:** Correlations between overall TIC profiles and histological characteristics of CXPAs.

histological characteristics	overall TIC profile type*		
	matrix-rich	cell-rich	intermediate
	(n = 4)	(n = 2)	(n = 2)
tumor encapsulation**			
invasive (≥1.5 cm)	0	1	2
non-invasive (<1.5 cm)	4	1	0
p-value****	0.050		
proportion of malignant component within tumor***			
dominant (≥75%)	1	1	2
not dominant (<75%)	3	1	0
p-value****	0.225		

*, CXPAs were categorized into 3 types (matrix-rich, cell-rich, and intermediate) based on the overall TIC profiles.

**, The tumor margins were categorized into invasive (tumor extension ≥1.5 cm beyond the capsule) or non-invasive (<1.5 cm).

***, The proportion of malignant components within the tumors were categorized into dominant (≥75% tumor areas were occupied by malignant components) or not dominant (<75%).

****, chi-sqiare test (two-tailed)

**Fig 4:**
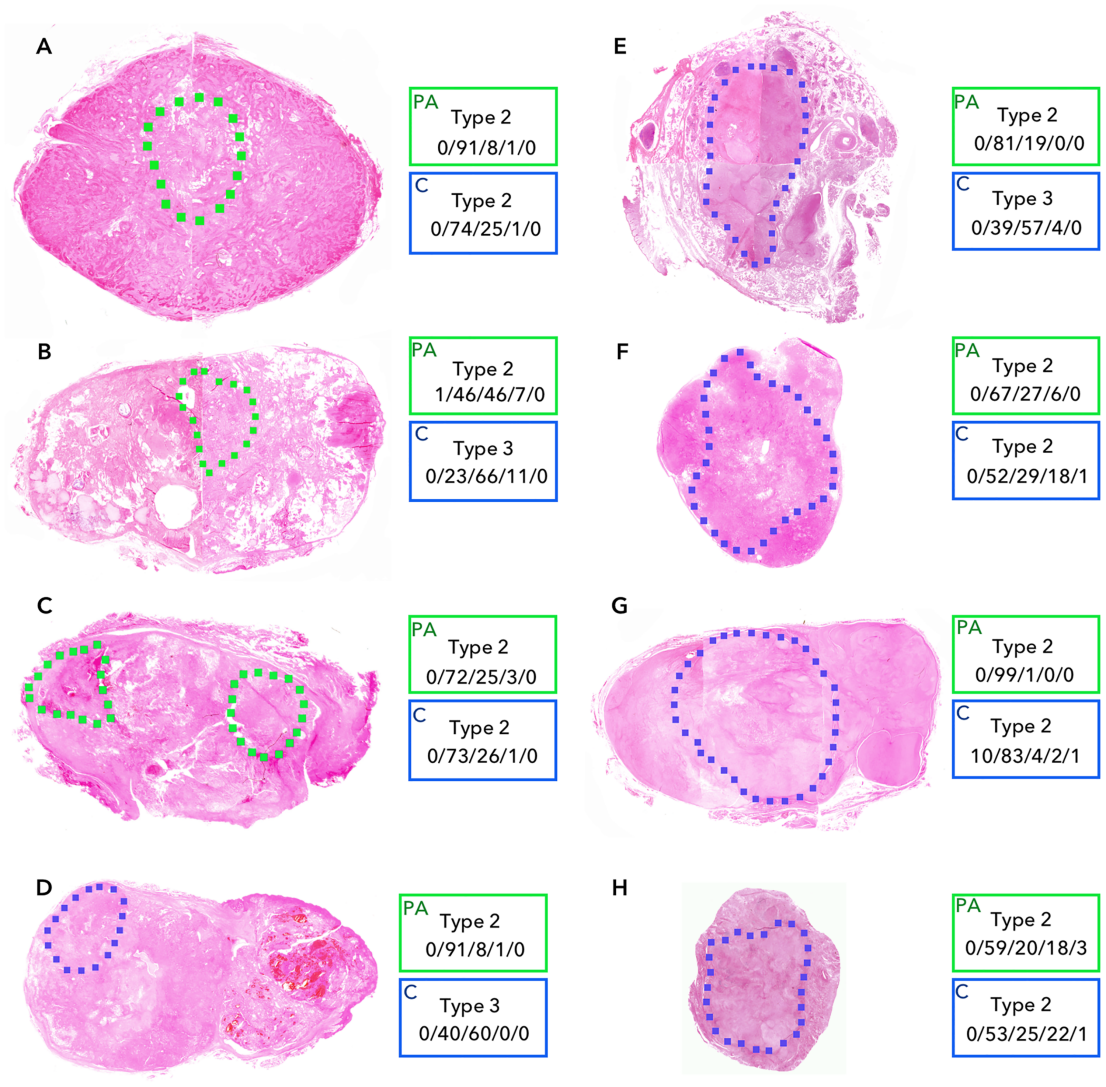
TIC profiles of pre-existing PA and malignant areas of 8 CXPAs. Overall TIC profile analysis and TIC mapping were performed separately in the pre-existing PA and malignant tumor areas in 8 CXPAs. Green and blue dashed lines on photomicrographs, respectively, indicate pre-existing PA and malignant tumor areas within CXPA tumor areas from 8 patients (**A**-**H** for patient no. 1–8 in the same order; see Table 2). Boxes directly right to the respective photomicrographs (H & E staining) show overall TIC profiles (upper panel in each box) and TIC mapping (lower panel in each box; % tumor areas as Type 1/Type 2/Type 3/Type 4/Type 5) of pre-existing PA (green, PA) and malignant tumor (blue, C) areas within CXPA tumors.

The progression from PA to CXPA is usually due to the emergence of an epithelial, malignant component within a pre-existing, usually highly hyalinized PA. The TIC patterns are based in the proportion of extracellular matrix versus cellular compartment. Therefore, we reasoned that the malignant progression is associated with TIC profile transition from Type 2 to Type 3 or 4 patterns. We found that the pre-existing PA areas exhibited Type 2 overall TIC profiles, while most of the malignant areas showed Type 3 overall TIC profiles or Type 2 overall TIC profiles with larger Type 3/4 TIC tumor areas compared with the corresponding pre-existing PA areas (Fig 4).

## Discussion

In the present study, we aimed to differentiate CXPA from PA using pixel-based TIC analysis. The stepwise approach with multiple TIC cut-off thresholds allowed us to differentiate CXPAs from PAs with 75% sensitivity, 95% specificity, 86% accuracy, 86% positive and 90% negative predictive values. The obtained diagnostic ability was higher than that with a single use of TIC thresholds. However, the diagnostic accuracy remained less than 90% and 3 out of the 8 CXPA cases were misdiagnosed using the stepwise approach.

The TIC analysis has been most frequently used for assessing DCE MR images of salivary gland tumors. In a TIC, the early slope phase corresponds to the contrast agent transfer to the tissues, and it is considered to be largely dependent upon the blood flow rate, inflow blood volume, and capillary permeability [8]. The magnitude of the maximum signal intensity is representative of the volume of the extravascular-extracellular space, whereas the maximum time is related to the extravascular-extracellular space per contrast agent transfer to the tissues. Although the capillary density and blood volume of the tumors are closely related to the kinetics of contrast enhancement, cellularity can also contribute to the early slope phase. In cancers, for example, high capillary permeability and poor lymphatic drainage create a condition of limited cellularity [12]. Low cellularity may lead to slow uptake of the contrast agent. Meanwhile, the washout ratio depends upon the histological type, such as phenotypes of the cancer cells and properties of the cancer stroma. Therefore, the mechanisms that influence the washout ratio of the contrast agent in these tumors are complex. Contrast enhancement patterns can also be greatly influenced by the vascular permeability of the lesions. Capillary density may be a minor physiologic factor compared to vascular permeability with regard to the differences in TIC profiles [13, 14].

In a pixel-based TIC analysis, cystic/necrotic areas display Type 1 TIC patterns and cancer nests containing loosely packed cancer cells associated with extensive fibrotic/myxoid tissues may have Type 2 or Type 3 TIC patterns depending on the density of cancer nests and fibrosis. Tumor areas with densely packed cancer cells are often associated with Type 4 TIC patterns [15, 16]. The cancer focus area in CXPA varies greatly from one tumor to another. In addition, PAs also have histologic features with a wide variety of benign components [4]. Therefore, a single TIC criterion is not sufficient for the effective differentiation of CXPA from PA. The stepwise approach with multiple TIC indices permitted effective differentiation of CXPAs from PAs. The overall profiles of CXPAs can be categorized into either of the 3 types with varying combinations of tumor areas of distinctive TIC profiles; these included (a) cell-rich type with ≥15.0% Type 4 areas, (b) matrix-rich type with ≥60% Type 2 areas, and (c) intermediate type with <60% Type 2 and <15% Type 4 areas. However, PAs had an additional TIC type with ≥50% Type 1 areas (cystic type). The present study findings implied that the intermediate type with rich cancer nests and matrix might represent a more aggressive form of CXPA. This is consistent with the previous report that aggressively growing cancer foci in metastatic nodes exhibited Type 2 and Type 3-dominant TIC profiles [15]. Furthermore, comparison of TIC profiles between pre-existing PAs and malignant tumor areas within CXPAs in the present study supports the notion that malignant transformation from the pre-existing PA is associated with a transition of the tumor perfusion characteristics (from Type 2 to Type 3/4 TIC profiles). However, any causal relationship was found between the false negative results of the 3 patients (no. 2, 5, and 7) and the TIC profiles of the pre-existing PA and malignant tumor areas (see Table 2 and Fig 4B, 4E and 4G).

The fact that 3 cases out of the 8 CXPA patients were misdiagnosed using the stepwise differentiation may be a very critical problem for the preoperative imaging diagnosis. In addition, the false-negative CXPA cases had no consistent TIC or histological characteristics in the present study. The poor prognostic factors for CXPA reportedly included extracapsular extension of cancer components >2.5 mm from the capsule of the preexisting PA [6]. However, the detection of extracapsular extension using MR imaging can be very challenging; in fact, the morphology of the tumor margin and capsule was indistinguishable between CXPA and PA. In the present study, we used a higher threshold of 1.5 cm for the invasive nature of the tumor [11], and found that the overall TIC profiles moderately correlated with the tumor encapsulation characteristics. Another prognostic factor is the presence of myoepithelial carcinoma in CXPA [17]. The clinical behavior of minimally or non-invasive CXPA is not aggressive compared to that of widely invasive CXPA [5, 18]. However, the presence of myoepithelial carcinoma subtype within a minimally invasive CXPA may increase the risk of recurrence [17]. In contrast, Lewis et al. denied the prognostic importance of histological subtypes in malignant components [11]. In the present study, we did not find any obvious correlations between the histological subtypes of malignant components and the TIC profiles. However, another study with a large cohort of CXPA patients is needed to confirm this finding.

A major limitation of this study is that the diagnostic ability achieved via the stepwise differentiation with multiple TIC indices was not sufficiently high, and 3 out of 8 CXPAs were misdiagnosed using this approach. Related to this shortcomings, CXPA is an infrequent tumor and higher numbers are difficult to achieve. The subdivision into further categories, both of TIC patterns and histological features leads to very low numbers in each category. Therefore, small numbers of false positive/negative cases greatly affect the results. Water diffusion is the physiological property that can be predicted separately from tissue perfusion on diffusion-weighted MR imaging depending upon the b-values used [19]. Incorporation of the tumor diffusion characteristics in the criteria could achieve further improvement in the diagnostic ability for differentiating between CXPAs and PAs. The diffusion coefficient of salivary gland tumors used as as a single criterion or combined with TIC characteristics can effectively distinguish between benign and malignant tumors [16, 20, 21]. Furthermore, diffusion coefficient values were found to correlate well with the histological types of salivary gland tumors [10]. This hypothesis should also be evaluated further in a future study.

The multi-step differentiation system using different TIC patterns may be boring for physicians and radiologists, and different TIC cut-off thresholds may be needed for a different patient cohort. However, the present study has confirmed the usefulness of the computer-assisted detection/differentiation algorithms (TIC and cluster analyses) in differentiating CXPA from PA that had complex and similar perfusion MR imaging characteristics. These results suggested that the machine learning algorithm would be promising for the effective diagnosis of salivary gland tumors with incredibly complex and multi-dimensional medical imaging patterns that exceed human pattern recognition capabilities [22].

## Conclusions

In this study, we showed that stepwise differentiation using multiple TIC cut-off thresholds differentiated CXPA from PA with 75% sensitivity, 95% specificity, 86% accuracy, and 86% positive and 90% negative predictive values. It should be noted that the obtained diagnostic ability cannot be guaranteed in a different patient cohort. However, at present we have no imaging tools that has been reported to be promising for the preoperative diagnosis of CXPA in a patient with long-standing PA, other than MR imaging. Estimating the perfusion property may be effective for predicting CXPA and be useful for planning the management of patients with long-standing PA.
